# Integrating Plyometric and Flexibility Exercises via Traditional Games in Youth Rhythmic Gymnastics: A Randomized Controlled Trial

**DOI:** 10.3390/children13070884

**Published:** 2026-06-30

**Authors:** Ghazi Racil, Stefano Vando, Johnny Padulo, Domenico Martone

**Affiliations:** 1 Department of Biological Sciences, Higher Institute of Sport and Physical Education of Gafsa, Gafsa 2100, Tunisia; ghazi_racil@yahoo.fr; 2 Research Laboratory (LR 23JS01) “Sport Performance, Health & Society” Higher Institute of Sport and Physical Education of Ksar Said, Tunis 2086, Tunisia; 3 Fitkion—Center for Kinesiology and Adapted Physical Activity, 04100 Latina, Italy; stefano.vando@gmail.com; 4 Department of Biomedical Sciences for Health, Università degli Studi di Milano, 20133 Milan, Italy; 5 Department of Economics, Law, Cybersecurity and Sports Sciences, University Parthenope, 80035 Naples, Italy; domenico.martone@uniparthenope.it

**Keywords:** muscles, strength, flexibility, adherence, physical performance, games

## Abstract

**Highlights:**

**What are the main findings?**

- Embedding plyometric or flexibility drills within traditional games produced modality-specific adaptations: plyometric games (PECG) most improved squat- and countermovement jump performance, agility and dynamic balance, whereas flexibility games (FECG) most improved hip range of motion.
- The 8-week, game-based program was completed with full session attendance, no dropouts and no injuries.

**What are the implications of the main findings?**

- Coaches can use culturally familiar traditional games as an engaging vehicle to deliver targeted plyometric or flexibility stimuli to young rhythmic gymnasts, choosing the game type according to the physical quality they wish to develop.
- As benefits were training-specific and some effect sizes were modest, game-based drills should complement—rather than replace—sport-specific conditioning, and larger confirmatory trials are warranted.

**Abstract:**

**Background/Objectives**: Standard plyometric, flexibility and balance drills are central to youth rhythmic gymnastics but can be limited by repetitive monotony and reduced engagement. This study evaluated whether embedding plyometric or flexibility exercises within traditional games improves physical performance and lower-limb flexibility in young female gymnasts. **Methods**: Forty-two female gymnasts (age 13.7 ± 1.5 years) were randomly assigned (1:1:1, permuted-block randomization with allocation concealment) to a game-based plyometric group (PECG), a game-based flexibility group (FECG), or a standard gymnastics control (GE). Over 8 weeks (five sessions per week) the experimental groups performed their protocols immediately before regular training, with load recalibrated every two weeks. Squat jump (SJ), countermovement jump (CMJ) and hip flexibility were pre-specified primary outcomes; agility, Stork balance and Y-Balance were exploratory. Linear mixed models tested Group × Time interactions, with within-group effect sizes expressed as Cohen’s d. **Results**: Significant Group × Time interactions emerged for the squat jump (*F*_2,39_ = 6.09, *p* = 0.005) and countermovement jump (*F*_2,39_ = 6.77, *p* = 0.003), favoring PECG (SJ β = 1.90; CMJ β = 1.86). FECG produced the largest flexibility gains, with significant Group × Time interactions for hip abduction and left hip flexion; a marked within-group increase in right hip extension (β = 2.65) was not accompanied by a significant interaction and is interpreted with caution. PECG also improved agility (*p* = 0.001), static balance and several Y-Balance directions. No injuries or dropouts were recorded. **Conclusions**: An 8-week game-based intervention enhanced physical performance in adolescent rhythmic gymnasts, with effects largely specific to the training modality: plyometric games improved explosive power, agility and dynamic balance, whereas flexibility games improved range of motion. Given the small sample, the findings should be regarded as preliminary and hypothesis-generating.

## 1. Introduction

Rhythmic gymnastics is an artistic athletic discipline that combines gymnastic skills with dance-like movements, emphasizing both physical prowess and aesthetic appeal. Gymnasts demonstrate agility, flexibility, and coordination while manipulating apparatuses in harmony with music, creating a visually captivating display of strength and artistry [1].

Physical training is fundamental to the development of a rhythmic gymnast, as it provides the foundation necessary to execute complex routines with both precision and grace [2]. While children naturally acquire basic motor skills as they mature, mastering the technical demands of this sport requires a carefully cultivated environment to foster their potential [3].

Strength is fundamental in rhythmic gymnastics, underpinning the sport’s characteristic movements [4]. Plyometric training—which utilizes rapid eccentric pre-stretching followed by explosive concentric contractions—enhances power output through the storage of elastic energy [5]. This method is essential for developing the relaxation, explosive strength, and power required for complex gymnastics maneuvers. Beyond its technical value, plyometric training is effective across diverse populations, successfully optimizing performance in both clinical and athletic groups [6], and in recent studies this training method’s effectiveness and safety were confirmed [7,8]. Consequently, strength and power development remain essential components of a gymnast’s progression [9].

Flexibility—the range of motion in joints and the suppleness of tissues—improves with musculoskeletal growth and is vital for elite gymnastics [10]. This quality demands early, intensive training to facilitate enhanced jumps and complex maneuvers, as it correlates strongly with power, balance and coordination [11]. Consequently, training programs should target motor performance alongside specific strength, jumping, and flexibility markers [4].

Postural balance is a complex process shaped by biomechanical, neurophysiological, and cognitive factors [12,13]. Essential for athletic performance and injury prevention, balance training is critical across all stages of long-term development [14]. In rhythmic gymnastics, it fosters stability, optimizes afferent sensory feedback, and enhances motor skill proficiency [15]. From another side, agility—the ability to perform rapid, precise changes in velocity or direction—is equally fundamental [16]. Consistent, long-term interventions (4 to 48 weeks) have been shown to significantly improve these physical attributes and muscle power in young gymnasts [17,18].

While standard protocols like plyometrics, flexibility and balance drills are essential for gymnastics, their efficacy can be limited by repetitive monotony. Conversely, traditional games provide a dynamic environment that mandates high-level neuromuscular engagement through unpredictable shifts in intensity and direction, fostering superior neural plasticity and movement adaptability [3,15]. Furthermore, addressing motivation is critical; in rhythmic gymnastics, this is intrinsically linked to the coach–athlete relationship, where inadequate support can lead to psychological imbalances [19]. Traditional games play a crucial role in childhood development by establishing the motor foundations necessary for complex athletic progression and overall fitness [3,15]. Despite their clear potential for rhythmic gymnastics, scientific evidence remains scarce regarding the extent to which these games can augment the outcomes of standard youth training programs.

Given the limited research on factors enhancing lower-limb flexibility and plyometric-induced jump performance in young female rhythmic gymnasts, we conducted an eight-week controlled study. Our research evaluates an alternative pedagogical approach—embedding conventional plyometric and flexibility exercises within traditional game structures—to determine their impact on physical performance and lower-limb flexibility.

We hypothesize that an 8-week game-based training program will yield significantly greater improvements in reactive agility and vertical jump power in young gymnasts compared to those undergoing traditional, repetitive drills.

## 2. Materials and Methods

### 2.1. Participants

Forty-two female gymnasts (age 13.7 ± 1.5 years, body mass 39.3 ± 2.9 kg, stature 1.47 ± 0.04 m) were recruited from three local regional gymnastic teams and volunteered to take part in this study (Table 1). Before the study began, we ensured that the researchers involved in participant recruitment were blinded to the group assignment until the participants were officially enrolled. All participants were randomly assigned to one of three groups: a traditional game (PECG, *n* = 14) practicing plyometric exercises combined with gymnastics, a flexibility group (FECG, *n* = 14) combining flexibility exercises with gymnastics, or a control group (GE, *n* = 14) training only in gymnastics. Participants were instructed not to change their dietary or lifestyle habits during the intervention period. The three groups were compared to determine the effects of integrating flexibility-focused and strength-focused traditional games into gymnastic training programs.

Prior to the start of the study, written informed consent and assent were obtained from the participants’ parents or legal guardians and the participants themselves, respectively, in accordance with international ethical standards and the 1964 Helsinki Declaration and its later amendments. Following this, the experimental procedures were explained to all participants in age-appropriate language, and they were told that they could withdraw from the study at any time. This study was approved by the institutional research ethics committee.

Inclusion criteria required participants to have at least five years of continuous, active involvement in their team, with no lower extremity injury in the past 12 weeks. Additionally, none of the gymnasts had been involved in a regular plyometric training program for three months prior to this study.

### 2.2. Physical Fitness Evaluation

Flexibility was evaluated before and after the 8-week intervention period. Hip joint measurements were performed after a 10 min warm-up using a two-arm Jamar universal goniometer (Jamar Stainless-Steel Goniometers; Lafayette Instrument, Lafayette, IN, USA) to assess the range of motion [20]. This portable device has good to-excellent reliability [21]. Abduction, flexion and extension of the right and left legs were measured in degrees. To perform the measurements, the participant lay in a recumbent position and performed a slow, maximal active abduction of one leg while the other was held in straight extension, followed by a slow, maximal active flexion of each leg. Finally in the prone position, participants performed a slow, maximal active extension of each leg.

Squat jump (SJ) and countermovement jump (CMJ) performance were measured using an infrared jump system (Optojump, Microgate^®^, Bolzano, Italy) interfaced with a computer. The Optojump system operates at a sampling frequency of 1000 Hz, and its validity and measurement error for flight-time-based jump height have been documented previously [22]. A trial was considered invalid if any premature knee flexion was detected at the start of the SJ. For the CMJ, participants started from an upright standing position and performed a downward movement by flexing the knees and hips to approximately 90°. A trial was considered invalid if the countermovement was not performed quickly or if the depth was insufficient [23]. Verbal instructions were provided for all three trials, and the best performance was recorded for analysis.

The Stork Balance Test (SBST) was performed as described by [24]. The test was conducted on the dominant leg under two conditions: eyes open (OEB) and eyes closed (CEB). Participants completed three trials for each condition, and the longest duration of maintained balance was recorded. Subjects stood with the non-supporting foot pressed against the medial aspect of the supporting knee, with hands placed on their hips. On command, participants lifted their heel off the ground and maintained the position for as long as possible. Timing commenced when the heel was lifted and concluded if the hands left the hips, the supporting foot moved or the heel touched the ground. To optimize results, participants completed familiarization trials prior to data collection.

Each participant performed three attempts, and the longest duration maintained was recorded for analysis. The previously reported test–retest reliability scores for OEB and CEB were 0.84 and 0.79, respectively, with 95% confidence intervals of 0.54–0.85 and 0.58–0.86; these coefficients are taken from the earlier literature for this test and are distinct from the test–retest reliability calculated on the present sample, which is reported in the Statistical Analysis section (Section 2.4).

The Y-Balance Test (YBT) was used to measure dynamic balance. Participants performed the reach in the following order: right anterior, left anterior, right posteromedial, left posteromedial, right posterolateral and left posterolateral. During the test, participants balanced on one leg while reaching as far as possible with the opposite leg in each of the three directions. To ensure accuracy, each direction was tested multiple times, and the maximum reach distance was recorded to the nearest 0.5 cm. This test is widely recognized for assessing dynamic stability and neuromuscular control [25].

Participants perform three successful consecutive trials on one limb before repeating the procedure on the contralateral side. Once three successful reaches are completed in each direction for both feet, they progress to the next direction. The administrator records each reach distance to calculate the participant’s YBT composite score. The *t*-test of agility was performed as described by [24]. This widely used assessment measures an athlete’s ability to change direction while running. A trial is invalidated if the participant crosses their feet, fails to touch the base of the cones with their leading hand, or does not maintain a forward-facing posture. The test is timed using photocells, and the final score is the fastest of three successful trials. The photocells employed for timing resolution 0.01 s, constitute an instrument whose reliability and criterion validity for repeated change-of-direction performance have been reported [26]. To ensure reliability, all participants completed one familiarization session 48–72 h prior to baseline testing, utilizing the same equipment, verbal instructions, and assessor procedures as the formal testing sessions.

### 2.3. Training Program

The GE group completed their standard routine of five gymnastic sessions per week. Over an 8-week period, the PECG and FECG groups performed their respective training protocols—plyometrics or flexibility—immediately preceding their regular gymnastics training. Thus, all three groups followed the same weekly schedule of five sessions per week, so that the total weekly training exposure was comparable across groups (Table 1); the plyometric or flexibility content of the experimental groups was embedded within these existing sessions rather than added as additional weekly sessions. Each session consisted of a 10 min warm-up, a 25 to 40 min intervention phase, 60 to 75 min of sport-specific gymnastics training, and a 10 min cool down (stretching). During the intervention phase, participants engaged in traditional games adapted to facilitate progressive neuromuscular and flexibility demands. To ensure progressive overload, training load parameters were recalibrated every two weeks (Table 2). Training intensity and session duration reached their peak during the final session (Table 3).

#### 2.3.1. Jumping Squares (PECG)

This game required unilateral and bilateral hopping patterns, evolving from foundational jumps to complex explosive movements, including burpees and tuck jumps, to enhance reactive power and neural drive.

#### 2.3.2. Sidi Bou Saïd Steps (PECG)

A staircase-based protocol utilizing a series of movements—including high knees, lateral, forward, and backward jumps—designed to develop explosive lower-limb strength and coordination. Detailed training load parameters for each ‘Jumping Squares’ and ‘Sidi Bou Saïd’ Steps, including total sets, repetitions, and prescribed rest intervals, are provided in Table 2. These variables were recalibrated every two weeks to maintain an optimal progressive overload stimulus, accounting for potential adaptations across the 8-week study period.

#### 2.3.3. Flexibility Protocol (FECG)

Exercises included guided stretching sequences (e.g., Pigeon Pose, Extended Side Angle, and Half-Moon Pose) and a Limbo-based game, which utilized rhythmic movements to improve joint range of motion and proprioceptive control. It is worth noting that flexibility exercises were performed with similar session volume progression, focusing on increased hold duration and range of motion intensity throughout the 8 weeks.

### 2.4. Statistical Analysis

Data are expressed as mean ± standard deviation (SD). Normality of distribution and homogeneity of variance were verified using the Shapiro–Wilk and Levene’s tests, respectively. Following enrollment and informed consent, participants were assigned to one of three study groups in a 1:1:1 ratio. The random allocation sequence was generated after baseline testing by an independent investigator using SPSS (v.26.0, IBM, Chicago, IL, USA) via permuted block randomization. To ensure allocation concealment, group assignment was delivered through a password-protected, web-based system accessed only after official enrollment. To examine the effects of the training interventions, linear mixed models (LMMs) were fitted for each dependent variable, including Group (PECG, FECG, GE) and Time (Pre, Post) as fixed effects, and their interaction (Group × Time), with GE serving as the reference category. For each group, the β coefficient reported in Table 4 and Table 5 represents the estimated within-group change from pre- to post-intervention (i.e., the simple effect of Time within that group), expressed in the measurement unit of each outcome; it does not represent a between-group contrast. The omnibus F-statistic reflects the Group × Time interaction, and a statistically significant interaction was the primary basis for inferring a differential training effect between groups. Reliability was assessed using the Intraclass Correlation Coefficient (ICC) and the Coefficient of Variation (CV%). Test–retest reliability was excellent (ICC = 0.94; 95% CI: 0.88–0.97), and absolute reliability analysis revealed a CV% of 3.2%, indicating minimal measurement error between testing sessions. This value represents the overall (pooled) test–retest reliability computed across the outcome measures collected in the present sample, rather than a separate coefficient estimated for each individual test; reliability values cited for specific tests in the Methods section (e.g., the Stork Balance Test) are literature-derived and were not re-estimated on the present sample. Significant Group × Time interactions were interpreted as differential training effects. Within-group pre-to-post changes were further assessed using paired samples *t*-tests, with within-group effect sizes reported as Cohen’s d (calculated as the mean pre-to-post change divided by the pooled pre/post standard deviation) and 95% confidence intervals (CI). Effect sizes were interpreted as trivial (<0.20), small (0.20–0.49), medium (0.50–0.79), and large (≥0.80)) [27]. An alpha level of 0.05 was adopted for all analyses. A formal a priori power analysis was not performed, because the available sample was constrained by the number of competitive youth rhythmic gymnasts accessible within the participating clubs; the sample size therefore follows a resource-constraint justification. For transparency, a sensitivity analysis indicated that, with fourteen participants per group, an alpha of 0.05, and a statistical power of 0.80, the smallest effect the design could reliably detect corresponded to a standardized mean difference of approximately d = 1.10 for pairwise contrasts and a Cohen f of approximately 0.50 for the omnibus Group by Time effect; smaller effects may therefore have gone undetected, and the findings are accordingly interpreted as preliminary and hypothesis-generating. Vertical jump performance (squat jump and countermovement jump) and hip flexibility were pre-specified as the primary outcomes, given their direct relevance to rhythmic gymnastics, whereas agility, static (Stork) balance and the individual Y-Balance Test directions were treated as exploratory. Because the primary outcomes were few and pre-specified, no formal correction for multiple comparisons was applied; instead, exact *p* values, effect sizes and 95% confidence intervals are reported for every outcome so that each result can be weighed on its own merits.

## 3. Results

The participants in the PECG, FECG and the GE groups took part in all training sessions. No injuries were reported during the intervention and in terms of compliance; no participants withdrew from the study. No significant difference appeared between the groups in the body mass data at baseline, and neither group showed a notable change in body mass (*p* ˃ 0.05, Table 4).

The training groups responded to the conditions of the training program as assigned. The LMM analysis revealed significant Group × Time interactions for most physical performance and flexibility variables, indicating that training adaptations were specific to the intervention modality. Regarding vertical jump performance, significant group × time interactions were observed for both the squat jump (*F*_2,39_ = 6.09, *p* = 0.005) and the countermovement jump (*F*_2,39_ = 6.77, *p* = 0.003). Post hoc analyses confirmed that the plyometric-based group (PECG) achieved superior explosive performance, with significant improvements in SJ (β = 1.90, SE = 0.21, 95% CI [1.49, 2.31]) and CMJ (β = 1.86, SE = 0.30, 95% CI [1.27, 2.45]). For lower-limb flexibility, significant interactions were found for parameters such as left hip flexion (*F*_2,39_ = 3.34, *p* = 0.046). The FECG protocol demonstrated notable improvements in hip flexibility, particularly in right hip extension (β = 2.65, SE = 0.80, 95% CI [1.08, 4.22]) and left hip extension (β = 1.67, SE = 0.60, 95% CI [0.49, 2.85]). It should be noted that, although these within-group increases in hip extension were sizeable, the corresponding Group × Time interactions did not reach statistical significance (right hip extension *p* = 0.180; left hip extension *p* = 0.915); they are therefore reported as within-group changes rather than as evidence that FECG was superior to the other groups for hip extension. On another side, significant group × time interactions were also identified for agility (*F*_2,39_ = 8.29, *p* = 0.001) and postural stability measured by the Stork Balance Stand Test (SBST). Specifically, for SBST (OE), the PECG group showed significant improvement (β = 3.20, SE = 0.42, 95% CI [2.37, 4.03], *p* < 0.001). Indeed, the dynamic balance assessment revealed significant group × time interactions in multiple directions of the Y-Balance Test (Table 5). The PECG protocol was particularly effective in enhancing dynamic postural control. Notably, in the R Anterior direction, the PECG group showed a β = 1.29 (SE = 0.35, 95% CI [0.60, 1.98], *p* < 0.001), while in the L Postero-Lateral direction, the improvement reached β = 2.31 (SE = 0.45, 95% CI [1.43, 3.19], *p* < 0.001).

## 4. Discussion

Our study evaluated the efficacy of an 8-week intervention program—incorporating either plyometric (PECG) or flexibility (FECG) exercises embedded with traditional games on the physical performance and lower limb flexibility of female gymnasts. Our findings suggest that integrating game-based protocols may induce specific physiological adaptations depending on the training modality, indicating that training effects are highly specific to the intervention type.

Linear mixed-model analysis of lower-limb flexibility revealed a differential response to the FECG protocol. While the model showed statistically significant improvements in hip abduction and left hip flexion (*p* < 0.05), the effect sizes remained modest (*d* = 0.36); nevertheless, the significant interaction effect observed in left hip flexion suggests that flexibility-focused, game-based exercises may effectively enhance range of motion. These results align with the existing literature which suggests that consistent, structured flexibility training is essential for meeting the aesthetic and biomechanical demands of rhythmic gymnastics [28]. Notably, the FECG protocol induced a more pronounced within-group adaptation specifically in right hip extension, with the LMM indicating a meaningful change; however, because the omnibus Group × Time interaction for right hip extension was not statistically significant (*p* = 0.180), this finding should be regarded as a within-group observation rather than as evidence that FECG was superior to the other groups for this measure. Interpreted cautiously, it may nonetheless suggest that the FECG structure might target the mechanical constraints of hip extension. In this context, the incorporation of games as flexibility exercises may have contributed to neuromuscular adaptations, while the repetitive dynamic stretches performed during these activities appear to have facilitated improvements in neuromuscular coordination [28]. It is worth noting that the 8-week intervention period appears sufficient to induce adaptations in both the PECG and FECG groups. These outcomes are consistent with previous research suggesting that regular stretching over 4–8 weeks can potentially increase range of motion. This may be attributed to a combination of structural changes in muscle tissue and nervous system adaptations, reflecting a complex interaction between musculoskeletal and neural mechanisms [29,30]. These findings may be particularly relevant for practitioners, as increased hip flexibility often appears to correlate with higher performance scores in gymnastics [31]. Furthermore, these findings seem to corroborate [32] who underscored the importance of range-of-motion management in rhythmic gymnastics for injury prevention and performance optimization. Given the modest effect sizes for hip abduction and left hip flexion, we recommend incorporating the FECG protocol as a preparatory or supportive drill to complement more intensive flexibility training.

Regarding jump performance, the LMM analysis indicates that the PECG protocol elicited significant improvements in explosive strength compared to the standard gymnastics (GE) and FECG groups. Specifically, the model showed significant gains in squat jump (β of 1.90, *p* = 0.005) and countermovement jump (β of 1.86, *p* = 0.003). These results suggest that plyometric-based games contribute to explosive power—a capacity critical for the technical and aesthetic demands of rhythmic gymnastics [33].

While we did not directly quantify neuromuscular mechanisms, these gains likely reflect improved stretch-shortening cycle efficiency, as observed in similar athletic populations [33]. Notably, our findings align with the literature, showing that resistance training can enhance explosive strength in young gymnasts (Table 4) without compromising flexibility; comparable neuromuscular and jump-performance gains following plyometric training have been reported in young athletes [18,34].

While the FECG protocol proved its effectiveness for static balance (*d* = 2.45 and 0.91), the dynamic Y-Balance yielded a smaller effect size (*d* ≈ 0.36), suggesting these benefits are secondary or foundational rather than transformative. Indeed, significant group × time interactions were observed, with the PECG group showing widespread improvements. These findings indicate that plyometric exercises contribute not only to power but also to dynamic postural control, an essential quality for complex gymnastic elements. This nuance highlights that game-based integration provides an optimal balance for inducing neuromuscular adaptations and managing training loads. Training intensity in rhythmic gymnastics has likewise been shown to elicit measurable physiological responses in young gymnasts, underscoring the relevance of structured load management in this discipline [35,36]; it should complement, rather than replace, targeted, sport-specific mobility protocols. It is worth noting that our 8-week intervention demonstrated that game-based plyometrics, such as the ‘Sidi Bou Said’ exercise, serve as effective, engaging tools for enhancing explosive power. These findings align with broader evidence linking high-impact activities to improved bone density in adolescent girls and positive cognitive and motor outcomes in children [37].

Regarding agility performance, the LMM analysis revealed a significant group × time interaction (*p* = 0.001), with the PECG protocol eliciting superior gains compared to the FECG and GE groups. The PECG group achieved a marked improvement in agility test times, with a substantial effect size of *d* = 4.67, highlighting the efficacy of plyometric-focused games in enhancing explosive, sport-specific movement. This within-group effect size is unusually large because it reflects a sizeable mean pre-to-post change relative to a very small between-participant standard deviation in agility times; it should therefore be interpreted with caution and should not be read as an estimate of the between-group treatment effect, which is more conservatively captured by the Group × Time interaction. In contrast, the FECG (*d* = 0.78) and GE (*d* = 0.38) groups demonstrated only small changes. The superior agility performance observed in the PECG indicates that the integration of repetitive, high-intensity jumps—as utilized in our game-based protocol—provides an effective stimulus for enhancing sport-specific agility in young rhythmic gymnasts. These findings further suggest that the specific nature of the PECG training, which mimics the rapid, multidirectional explosive movements inherent in gymnastics, directly contributes to the improved agility performance measured in this study.

While these agility and postural stability results are promising, these exploratory findings should be viewed as preliminary and warrant further investigation in larger, confirmatory trials. Additionally, it is worth noting that the game-based delivery of these exercises may have contributed to a higher level of participant engagement throughout the 8-week intervention, potentially supporting consistent training attendance and effort. Future studies may benefit from incorporating formal psychometric assessments to qualify the relationship between game-based training formats, participant engagement and performance outcomes.

Several limitations of the present study should be acknowledged. While our results show the efficacy of game-based training protocol at improving physical performance, these findings should be considered preliminary, and these results should be interpreted with caution given the modest sample size. This constraint is consistent with the recognized difficulty of achieving adequate statistical power in small athletic samples, and the estimates reported here should be regarded as preliminary [38]. Future studies with larger, more diverse cohorts are needed to confirm these effects and better define the magnitude of the practical impact. Furthermore, we acknowledge the absence of quantitative or qualitative data, such as validated psychological questionnaires, to formally assess participant motivation, enjoyment, or emotional state throughout the intervention. Also, the lack of body composition assessment prevented us from definitively isolating changes in lean muscle mass from total body weight.

Finally, a notable limitation of this study is the lack of objective assessment regarding the biological maturation status of the participants. Given the mean age of 13.7 years, the cohort likely includes individuals at varying stages of pubertal development. Puberty is associated with rapid hormonal changes and structural growth, which are known to influence strength, neuromuscular control, and flexibility independently of training interventions. Consequently, while our results demonstrate positive adaptations, the extent to which these changes were influenced by natural maturation processes—rather than the intervention itself—remains a potential confounding factor. Future investigations in this population should consider utilizing methods such as Tanner staging to better isolate the effects of training from developmental growth. The influence of biological maturation on physical-performance development in youth athletes has been emphasized previously, and maturity status should be accounted for in future controlled designs [38].

## 5. Conclusions

In conclusion, this eight-week program effectively enhanced physical performance in adolescent rhythmic gymnasts through the integration of plyometric and flexibility exercises into game-based training. The strongest evidence concerns the pre-specified primary outcomes (vertical jump performance and hip flexibility), whereas the agility, static balance and Y-Balance findings were exploratory and, given the small sample and the number of outcomes examined, should be regarded as hypothesis-generating rather than confirmatory. It is important to clarify that the efficacy of our intervention is grounded in the strategic integration of well-established plyometric and flexibility principles. While the fundamental exercises are not inherently new, the novelty of this approach lies in the cultural contextualization of the training. By embedding these conventional movements into traditional games, we aimed to bridge the gap between structured physical conditioning and participant engagement. While this study provides significant evidence for the short-term benefits of intervention, future research should utilize longitudinal designs to investigate the long-term sustainability of these physical adaptations.

## Figures and Tables

**Table 1:** Participant characteristics in the three groups.

Variables	PECG	FECG	GE
Chronological Age (years)	13.5 ± 1.4	13.9 ± 1.6	13.7 ± 1.5
Training Age (years)	6.2 ± 0.8	6.5 ± 0.9	6.3 ± 0.7
Weekly Training Volume (hours/week)	08–10	08–10	08–10
Competition Level	Regional	Regional	Regional

All values are shown as mean ± SD. PECG: plyometric exercises combined with gymnastics; FECG: flexibility exercises combined with gymnastics; GE: Gymnastic exercises.

**Table 2:** Training load parameters and progression scheme for each ‘Jumping Squares’ and ‘Sidi Bou Saïd’ games.

Phase (Weeks)	Game	Sets	Repetitions/Duration	Rest Interval (Between Sets)	Intensity Progression
1–2	Jumping Squares	3	10 jumps per circuit	60 seconds (s)	Light (fundamental skills)
3–4	Jumping Squares	4	15 jumps per circuit	60 s	Medium (squat integration)
5–6	Jumping Squares	4	20 jumps per circuit	45 s	High (burpees/single-leg)
7–8	Jumping Squares	5	25 jumps per circuit	45 s	Maximal (tuck jumps)
1–2	Sidi Bou Saïd	3	5 mins continuous	60 s	Light (climbing/walking)
3–4	Sidi Bou Saïd	3	6 mins continuous	60 s	Medium (high knees/side steps)
5–6	Sidi Bou Saïd	3	7 mins continuous	60 s	High (forward/backward leaps)
7–8	Sidi Bou Saïd	3	8 mins continuous	60 s	Maximal (zigzags/explosive)

**Table 3:** Overview of plyometric or flexibility training schedule over an 8 week-period. Five sessions per week.

Periods	(PECG) and (FECG) Warm-Up + (Plyometrics or Flexibility) + Choreography + Recovery		(GE) Warm-Up + Choreography + Recovery		Time/ Session
1–2 week	10 min	15 min + 60 min + 10 min	10 min	75 min+ 10 min	1 h 35 min
3–4 week	10 min	20 min + 65 min + 10 min	10 min	85 min + 10 min	1 h 45 min
5–6 week	10 min	20 min + 70 min + 10 min	10 min	90 min + 10 min	1 h 50 min
7–8 week	10 min	25 min + 75 min + 10 min	10 min	100 min + 10 min	2 h 00 min

Abbreviations: PECG: plyometric exercises combined with gymnastics; FECG: flexibility exercises combined with gymnastics; GE: Gymnastic exercises.

**Table 4:** Comparison in pre- and post-intervention programs in physical performance indicators and flexibility measures.

	Group	Pre	Post	F(df)	*p*-Value	ES	β (SE)	95% CI
Body mass (kg)	PECG	42.42 ± 3.21	42.50 ± 2.40	1.11 (2, 39)	>0.05	-	0.08 (0.35)	[−0.61, 0.77]
	FECG	43.24 ± 3.63	43.30 ± 3.22			-	0.06 (0.30)	[−0.53, 0.65]
	GE	43.61 ± 4.64	43.70 ± 3.75			-	0.09 (0.40)	[−0.70, 0.88]
R hip abd (°)	PECG	62.45 ± 4.10	63.90 ± 4.40	4.69 (2, 39)	0.015	0.39	1.45 (0.50)	[0.47, 2.43]
	FECG	63.05 ± 5.10	64.70 ± 5.60			0.46	1.65 (0.62)	[0.43, 2.87]
	GE	62.40 ± 4.30	62.60 ± 3.90			0.21	0.20 (0.45)	[−0.68, 1.08]
L hip abd (°)	PECG	58.95 ± 5.00	59.92 ± 3.66	3.62 (2, 39)	0.036	0.27	0.97 (0.40)	[0.18, 1.76]
	FECG	59.14 ± 4.21	60.42 ± 4.30			0.45	1.28 (0.55)	[0.20, 2.36]
	GE	59.24 ± 5.13	59.41 ± 4.80			0.22	0.17 (0.35)	[−0.52, 0.86]
R hip Flex (°)	PECG	134.40 ± 4.30	134.90 ± 4.07	1.32 (2, 39)	0.281	0.17	0.50 (0.45)	[−0.38, 1.38]
	FECG	133.95 ± 3.30	135.60 ± 4.95			0.38	1.65 (0.60)	[0.47, 2.83]
	GE	134.25 ± 4.10	134.80 ± 3.50			0.21	0.55 (0.50)	[−0.43, 1.53]
L hip Flex (°)	PECG	133.35 ± 4.30	134.92 ± 4.32	3.34 (2, 39)	0.046	0.27	1.57 (0.65)	[0.29, 2.85]
	FECG	133.05 ± 4.10	134.85 ± 3.15			0.34	1.80 (0.72)	[0.38, 3.22]
	GE	133.80 ± 3.80	134.10 ± 4.00			0.26	0.30 (0.50)	[−0.68, 1.28]
R hip Ext (°)	PECG	38.75 ± 5.40	38.90 ± 4.05	1.79 (2, 39)	0.180	0.36	0.15 (0.50)	[−0.83, 1.13]
	FECG	36.15 ± 4.68	38.80 ± 4.25			0.69	2.65 (0.80)	[1.08, 4.22]
	GE	37.20 ± 4.50	37.45 ± 4.30			0.26	0.25 (0.40)	[−0.53, 1.03]
L hip Ext (°)	PECG	37.25 ± 4.54	37.30 ± 4.63	0.09 (2, 39)	0.915	0.25	0.05 (0.45)	[−0.84, 0.94]
	FECG	35.27 ± 4.74	36.94 ± 4.65			0.51	1.67 (0.60)	[0.49, 2.85]
	GE	36.00 ± 4.32	36.25 ± 4.20			0.28	0.25 (0.40)	[−0.53, 1.03]
SBST(OE) (s)	PECG	21.36 ± 2.24	24.56 ± 1.43	9.03 (2, 39)	<0.001	1.68	3.20 (0.42)	[2.37, 4.03]
	FECG	20.37 ± 1.70	24.14 ± 1.35			2.45	3.77 (0.38)	[3.02, 4.52]
	GE	20.90 ± 1.62	21.85 ± 1.28			0.65	0.95 (0.35)	[0.26, 1.64]
SBST (CE) (s)	PECG	15.20 ± 2.24	16.18 ± 1.15	5.85 (2, 39)	0.006	0.55	0.98 (0.30)	[0.39, 1.57]
	FECG	16.30 ± 1.40	17.58 ± 1.42			0.91	1.28 (0.28)	[0.73, 1.83]
	GE	16.40 ± 1.22	16.67 ± 1.64			0.19	0.27 (0.35)	[−0.42, 0.96]
Agility (s)	PECG	12.20 ± 0.20	11.15 ± 0.25	8.29 (2, 39)	0.001	4.67	−1.05 (0.10)	[−1.25, −0.85]
	FECG	12.10 ± 0.60	11.75 ± 0.12			0.78	−0.35 (0.15)	[−0.64, −0.06]
	GE	11.90 ± 0.72	11.65 ± 0.60			0.38	−0.25 (0.12)	[−0.49, −0.01]
SJ (cm)	PECG	18.20 ± 1.05	20.10 ± 1.10	6.09 (2, 39)	0.005	1.76	1.90 (0.21)	[1.49, 2.31]
	FECG	17.75 ± 1.10	18.05 ± 0.82			0.31	0.30 (0.18)	[−0.05, 0.65]
	GE	18.70 ± 0.79	18.85 ± 1.04			0.16	0.15 (0.15)	[−0.14, 0.44]
CMJ (cm)	PECG	22.26 ± 2.30	24.12 ± 2.20	6.77 (2, 39)	0.003	0.83	1.86 (0.30)	[1.27, 2.45]
	FECG	21.78 ± 3.45	22.05 ± 3.16			0.08	0.27 (0.25)	[−0.22, 0.76]
	GE	21.20 ± 2.90	21.47 ± 3.45			0.09	0.27 (0.22)	[−0.16, 0.70]

**Note:** M ± SD = Mean ± Standard Deviation; F(df) = F-statistic (degrees of freedom); ES = Effect Size; β = regression coefficient from LMM; SE = Standard Error; 95% CI = 95% confidence interval. R (L)hip abd (°): Right or left hip abduction in degrees; R (L) hip Flex (°): Right or left hip flexion in degrees; R (L) hip Ext (°): Right or left hip extension; SBST(OE): Stork Balance Stand Test with open eyes in seconds; SBST (CE): Stork Balance Stand Test with closed eyes in seconds; SJ: Squat jump; CMJ: Countermovement jump.

**Table 5:** Comparison in pre- and post-intervention programs and in Y-Balance Test performance.

Variable	Group	Pre	Post	F(df)	*p*-Value	ES	β (SE)	95% CI
R Anterior (cm)	PECG	62.41 ± 6.70	63.70 ± 6.44	8.80 (2, 39)	<0.001	0.48	1.29 (0.35)	[0.60, 1.98]
	FECG	63.12 ± 7.64	63.65 ± 6.22			0.21	0.53 (0.28)	[−0.02, 1.08]
	GE	62.87 ± 6.42	63.15 ± 6.28			0.18	0.28 (0.30)	[−0.31, 0.87]
L Anterior (cm)	PECG	63.15 ± 5.06	64.85 ± 4.60	2.37 (2, 39)	0.107	0.37	1.70 (0.60)	[0.52, 2.88]
	FECG	63.14 ± 7.20	63.75 ± 4.54			0.28	0.61 (0.45)	[−0.27, 1.49]
	GE	63.40 ± 6.12	63.85 ± 6.80			0.31	0.45 (0.35)	[−0.24, 1.14]
R Post. Med (cm)	PECG	98.32 ± 9.62	100.47 ± 8.80	0.32 (2, 39)	0.727	0.47	2.15 (0.75)	[0.68, 3.62]
	FECG	97.98 ± 10.30	99.55 ± 9.54			0.39	1.57 (0.60)	[0.40, 2.74]
	GE	97.70 ± 9.53	99.10 ± 9.38			0.24	1.40 (0.55)	[0.32, 2.48]
L Post. Med (cm)	PECG	100.11 ± 8.22	102.04 ± 8.10	3.76 (2, 39)	0.032	0.51	1.93 (0.50)	[0.95, 2.91]
	FECG	99.97 ± 9.63	100.32 ± 6.30			0.29	0.35 (0.25)	[−0.14, 0.84]
	GE	100.45 ± 10.24	101.10 ± 5.40			0.26	0.65 (0.40)	[−0.14, 1.44]
R Post. Lat (cm)	PECG	102.21 ± 7.52	104.14 ± 9.30	4.33 (2, 39)	0.020	0.48	1.93 (0.55)	[0.85, 3.01]
	FECG	102.57 ± 9.35	103.04 ± 9.72			0.34	0.47 (0.30)	[−0.12, 1.06]
	GE	102.78 ± 9.64	103.25 ± 9.86			0.21	0.47 (0.25)	[−0.02, 0.96]
L Post. Lat (cm)	PECG	101.65 ± 8.12	103.96 ± 8.43	8.61 (2, 39)	<0.001	0.49	2.31 (0.45)	[1.43, 3.19]
	FECG	102.67 ± 9.85	103.43 ± 9.62			0.34	0.76 (0.30)	[0.17, 1.35]
	GE	101.52 ± 9.04	102.34 ± 9.87			0.17	0.82 (0.35)	[0.13, 1.51]

**Note:** M ± SD = Mean ± Standard Deviation; F(df) = F-statistic (degrees of freedom); ES = Effect Size (Within-Group); β = regression coefficient from LMM; SE = Standard Error; 95% CI = 95% confidence interval. Significant interaction effects (*p* < 0.05) indicate non-parallel progression across groups. RA: Right Anterior; LA: Left Anterior; R Post. Med: Right Posteromedial; L Post. Med: Left Posteromedial; R Post. late: Right Posterolateral; L Post. late: Left Posterolateral.

## Data Availability

The original contributions presented in the study are included in the article. Further inquiries can be directed at the corresponding author.
